# PDGFRα-Signaling Is Dispensable for the Development of the Sinoatrial Node After Its Fate Commitment

**DOI:** 10.3389/fcell.2021.647165

**Published:** 2021-06-10

**Authors:** Xi Zheng, Fengjiao Wang, Xiaoxiao Hu, Hua Li, Zhen Guan, Yanding Zhang, Xuefeng Hu

**Affiliations:** ^1^Fujian Key Laboratory of Developmental and Neural Biology & Southern Center for Biomedical Research, College of Life Sciences, Fujian Normal University, Fuzhou, China; ^2^Key Laboratory of Stem Cell Engineering Societ and Regenerative Medicine, School of Basic Medical Science, Fujian Medical University, Fuzhou, China

**Keywords:** *Pdgfrα*, Nkx2.5, sinoatrial node (SAN), development, conditional knockout

## Abstract

Palate-derived growth factor receptor α (Pdgfrα) signaling has been reported to play important roles in the cardiac development. A previous study utilizing *Pdgfrα* conventional knockout mice reported hypoplasia of the sinus venous myocardium including the sinoatrial node (SAN) accompanied by increased expression of Nkx2.5. This mouse line embryos die by E11.5 due to embryonic lethality, rendering them difficult to investigate the details. To elucidate the underlying mechanism, in this study, we revisited this observation by generation of specific ablation of *Pdgfrα* in the SAN by Shox2-Cre at E9.5, using a *Shox2*-*Cre*;*Pdgfrα^*flox/flox*^* conditional mouse line. Surprisingly, we found that resultant homozygous mutant mice did not exhibit any malformation in SAN morphology as compared to their wild-type littermates. Further analysis revealed the normal cardiac function in adult mutant mice assessed by the record of heart rate and electrocardiogram and unaltered expression of Nkx2.5 in the E13.5 SAN of *Pdgfrα* conditional knockout mice. Our results unambiguously demonstrate that *Pdgfrα* is dispensable for SAN development after its fate commitment in mice.

## Introduction

The mature sinoatrial node (SAN), composed of an *Nkx2.5*^–^ head and an *Nkx2.5*^+^ sinoatrial junction or tail, is a group of specialized cells with obvious heterogeneity located on the dorsal wall of the right atrium close to the entrance of superior vena cava, functioning as the primary cardiac pacemaker that regulate the rhythm of the heartbeat by spontaneously producing electrical impulses (Christoffels et al., 2006; Yamamoto et al., 2006; Liu et al., 2007; Liang et al., 2013). The first heartbeat is recorded in the left inflow tract region as early as embryonic day (E) 8.0-E8.5 in mice (Yi et al., 2012), then the pace-making activity is localized to the right inflow (Dyer and Kirby, 2009; Csepe et al., 2015). These pacemaker cells will contribute to the definitive SAN and become morphologically discernible at E10.5 and form the comma-like structure with the SAN-like action potential configurations at E12.5 (Virágh and Challice, 1982). Deficiencies in its pacemaking function leads to cardiac arrhythmias, cardiac arrest, sinoatrial exit block, and even sudden cardiac death (Durham and Worthley, 2002).

A number of genes, including *Hcn4* (hyperpolarization-activated cyclic nucleotide-gated channel subtype 4), *Tbx3, Shox2*, and *Nkx2.5*, work as cardiac markers and are critical for the development of SAN. For instance, mutation in *Shox2* results in a severely hypoplastic SAN due to ectopic *Nkx2.5* expression in the head of SAN (Blaschke et al., 2007; Espinoza-Lewis et al., 2009). *Nkx2.5* is expressed in the precardiac tissues as one of early cardiac markers and is a key transcription factor for the development of working myocardium (Lints et al., 1993; Moses et al., 2001). *Nkx2.5* mutant display early embryonic lethality due to heart defects. However, *Nkx2.5* is only expressed in the junction of a developing SAN but absent in the head because of the transcription repression by *Shox2* and *Tbx3* (Christoffels et al., 2006; Espinoza-Lewis et al., 2009; Ye et al., 2015a,b). Mice lacking *Nkx2.5* in the SAN junction adopt normal morphology but physiological dysfunction, indicating that *Nkx2.5* is essential for SAN function but is dispensable for SAN morphogenesis (Li et al., 2019).

Platelet-derived growth factor receptor (PDGFR), located on the surface of a wide range of cell types, is bound by certain isoforms of PDGF ligands and thereby becomes active in stimulating cell signaling pathways that elicit responses including growth, development, and differentiation. There are two types of PDGFR, α and β, functioning in the form of homodimer, α-α (termed as Pdgfrα) and β-β (termed as Pdgfrβ), or heterodimer, α-β (termed as Pdgfrα-β) (Heldin and Westermark, 1999). Each type of the dimmer plays its distinct physiological function and does not display functional redundancy among them (Rosenkranz and Kazlauskas, 1999). Pdgfrα signaling plays a critical role in cardiac development, including the development of primary cardiac muscle, venous, and mesenchymal myocardium (Bax et al., 2010). Deletion of *Pdgfrα* causes neurological stenosis-related malformations at the heart outflow tract, such as persistent truncus arteriosus, double outlet right ventricle, ventricular myocardial insufficiency, and abnormal pulmonary veins (Morrison-Graham et al., 1992; Schatteman et al., 1995).

A previous study using green fluorescent protein (GFP) as a reporter in a knock-in mouse line (*Pdgfrα^*GFP*^*) reported that *Pdgfrα* is markedly expressed at the venous pole in the mesocardium, the myocardium of the sinus venosus, the proepicardial organ, and the coelomic mesothelium in the E9.5 heart, as well as in the developing SAN at E10.5. Several cardiac malformations including the significantly smaller SAN accompanied by increased expression of *Nkx2.5* were further observed in *Pdgfrα* conventional knockout mouse line (Bax et al., 2010). Unfortunately, most *Pdgfrα^–/–^* mice, even with intraperitoneal injection of isoprenaline in maternal mice, suffer severe early embryonic lethality by E11.5 when crossed in C57/B6 background in our animal facility, preventing detailed assessment of cellular and molecular events contributing to the abnormal morphology and function of sinus node, atrium, and ventricle. Here we attempted to further address this question using a conditional *Pdgfrα* knockout mouse line *Shox2-Cre;Pdgfrα^*flox/flox*^*, in which *Pdgfrα* expression was specifically ablated in the myocardium of the sinus venosus at E9.5 when the Cre activity is initiated (Sun et al., 2013). Unexpectedly, our results showed that homozygous mutant mice exhibit not only normal SAN morphology with normal heartbeat rate and electrocardiogram, but also unaltered *Nkx2.5* expression pattern in the SAN, indicating dispensability of Pdgfrα signaling after E9.5 for the development and differentiation of the SAN.

## Materials and Methods

### Mouse Line

*Shox2-Cre*, *Pdgfrα^*flox/flox*^* and *Rosa26*^*mTmG*^ mice were reported previously and obtained from Jackson Laboratory (Soriano, 1997; Muzumdar et al., 2007; Sun et al., 2013). To specifically inactivate *Pdgfrα* in the SAN, we crossed the mice carrying *Shox2-Cre* with *Pdgfrα^*flox/flox*^* mice to generate *Pdgfrα* conditional knockout mouse line *Shox2-Cre;Pdgfrα^*flox/flox*^*. We also compound *Shox2-Cre* allele with *Rosa26*^*mTmG*^ reporter mice for the lineage tracing of Shox2-Cre positive cells. All animal experiments in this study were approved by The Fujian Normal University Institutional Animal Care and Use Committee.

### Histology and Immunofluorescence

Mouse embryos were harvested from timed pregnant females. Bodies of collected embryos were fixed in 4% Paraformaldehyde at 4°C for 24 h and further processed for paraffin embedding and sectioning at 7 μm. Slides were subjected to Hematoxylin/eosin staining for histology analysis or immunofluorescent staining as described previously (Hu et al., 2014). The mean fluorescence intensity of *Pdgfrα* expression in SAN was quantified by using ImageJ software (National Institutes of Health). Antibodies used for this study include anti-Pdgfrα (R&D, AF1062), anti-Hcn4 (Abcam, ab32675), anti-Nkx2.5 (Santa Cruz, sc8697), and anti-GFP (Abcam, ab13970).

### 3D Reconstruction

Images of consecutive H&E stained sections (7 μm) of SAN and surrounding tissues were captured, stacked, and loaded into Amira 6.0.1, in which subsequent alignment, segmentation, and 3D model generation were performed.

### Surface Electrocardiography

Adult *Shox2-Cre;Pdgfrα^*flox/flox*^* mice and their wild-type littermates were anesthetized with 1.5% isoflurane supplemented with O_2_ and placed in prone position on a Mouse Monitor S (Indus Instruments) to be recorded as reported previously (Ye et al., 2015a,b).

### scRNA-seq Data Analysis

The protocol of the E13.5 SAN scRNA-seq was described in a previous study (Li et al., 2019) and this dataset is accessible under the GEO series accession number GSE130461. The E16.5 SAN scRNA-seq raw data was downloaded from NCBI/GEO datasets under accession number GSE32658. The visualization of *Pdgfrα* and *Nkx2.5* distributions was obtained with Uniform Manifold Approximation and Projection (UMAP) embedding.

### Statistical Analysis

Statistical analysis on the volume measurement were performed with independent unpaired Student’s *t* test. All data of the volume measurements are presented as mean ± SEM. Graphics of statistical analysis were performed with GraphPad Prism 5.0 software. *P* < 0.05 was considered statistically significant.

## Results

### Expression of *Pdgfrα* in the Developing SAN

**FIGURE 1:**
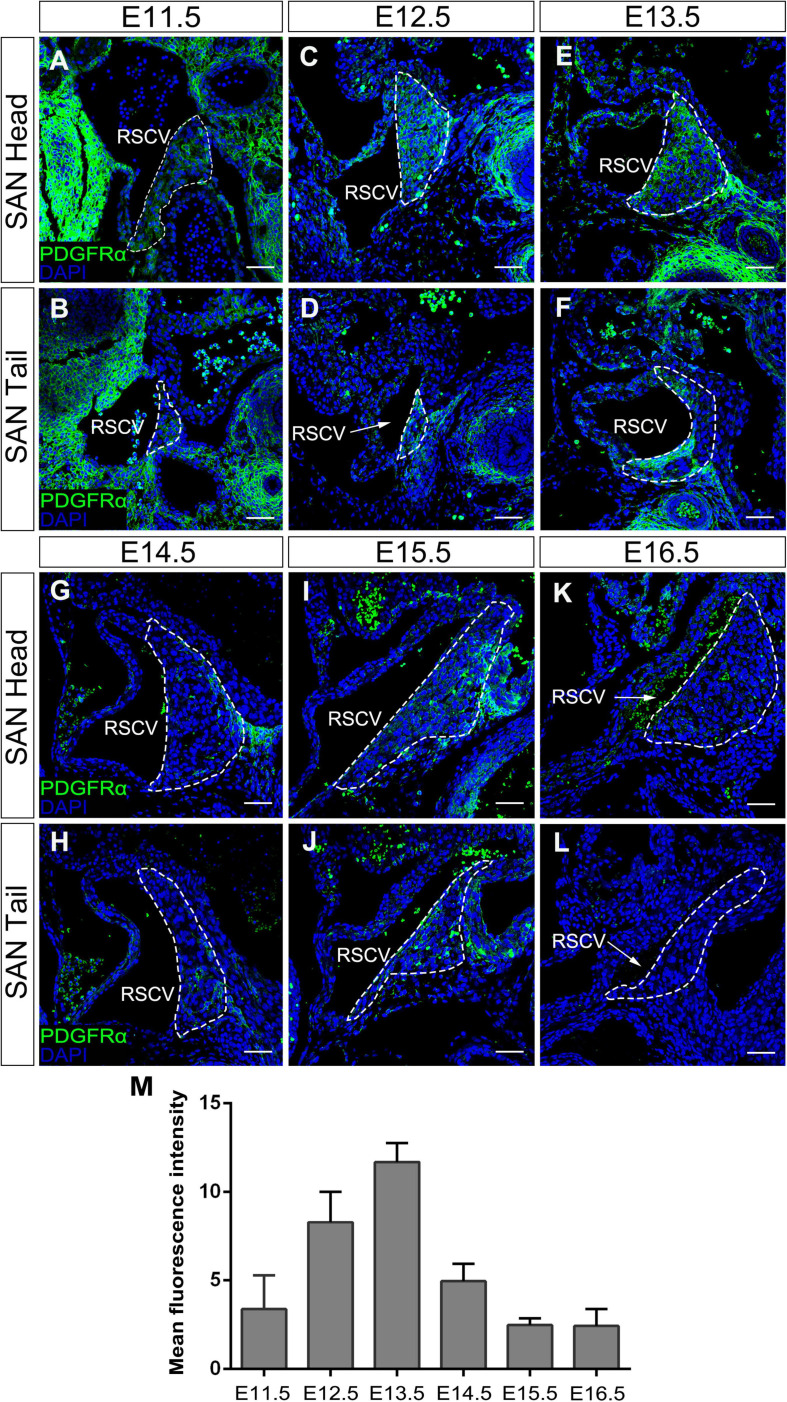
The expression pattern of *Pdgfrα* in the developing SAN. Immunostaining showing the expression of *Pdgfrα* in the head of the developing SAN (dotted lines) at E11.5 **(A)**, E12.5 **(C)**, E13.5 **(E)**, E14.5 **(G)**, E15.5 **(I)**, and E16.5 **(K)** and in the junction of the developing SAN at E11.5 **(B)**, E12.5 **(D)**, E13.5 **(F)**, E14.5 **(H)**, E15.5 **(J)**, and E16.5 **(L)**. **(M)** Semi-quantitative analysis of Pdgfrα fluorescent intensity in the developing SAN. RSVC, right superior vena cava. Scale bar = 100 μm.

It has been reported that *Pdgfrα* is expressed as early as E7.5 in several cardiac progenitor tissues including SAN during early heart development (Prall et al., 2007; Bax et al., 2010; Bloomekatz et al., 2017). To comprehensively document the *Pdgfrα* expression pattern during SAN morphogenesis, we further performed immunostaining of anti-Pdgfrα antibody in the developing SAN of wild-type mice from E11.5 to E16.5. Since the SAN is a structurally heterogeneous tissue composed of a head and junction with distinct gene expression profile (Li et al., 2019), we examined the expression of *Pdgfrα* in both domains, respectively. We found that, at E11.5, a weak expression of *Pdgfrα* was seen in both the head and junction of the SAN (Figures 1A,B). Whereas at E12.5 and E13.5, the expression of *Pdgfrα* reached to a relatively higher expression level in both of the domains (Figures 1C–F). At subsequent E14.5 to E16.5, *Pdgfrα* expression became dramatically decreased and manifested dim and sporadic pattern in the SAN (Figures 1G–L). By virtue of semi-quantitative analysis of fluorescence intensity at the different stage (*N* = 4/each stage), this pattern of *Pdgfrα* expression was confirmed (Figure 1M). Together, immunostaining data revealed that the expression of *Pdgfrα* in the developing SAN was weak at E11.5, then gradually upregulated to maximal levels at E13.5, and tapered to a faint level at E14.5 thereafter when the morphology and function of SAN is well established. In addition, *Pdgfrα* is evenly expressed in the whole SAN without obviously distinct pattern in both regions of the SAN. These observations implied that *Pdgfrα* may play a regulatory role during SAN development.

### No Morphological and Physiological Anomaly in the SAN of *Shox2-Cre*; *Pdgfrα^flox/flox^* Mice

**FIGURE 2:**
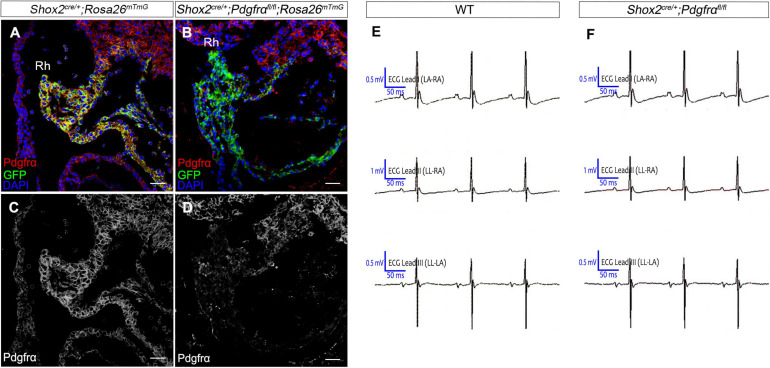
Normal cardiac function in *Pdgfrα* conditional knockout mice. **(A–D)** Co-immunostaining of Cre reporter mGFP and Pdgfrα verifies specific deletion of *Pdgfrα* in the E9.5 *Shox2-Cre*;*Pdgfrα^*flox/flox*^;Rosa26*^*mTmG*^ sinus venosus as compared to the positive staining in the *Shox2*^*cre/*+^*;Rosa26*^*mTmG*^ controls. **(E,F)** Representative recording of electrocardiogram (ECG) reveals normal cardiac function from *Pdgfrα* conditional knockout mice compared to that from the wild-type. Rh, right horn of sinus venosus. Scale bar = 50 μm.

**FIGURE 3:**
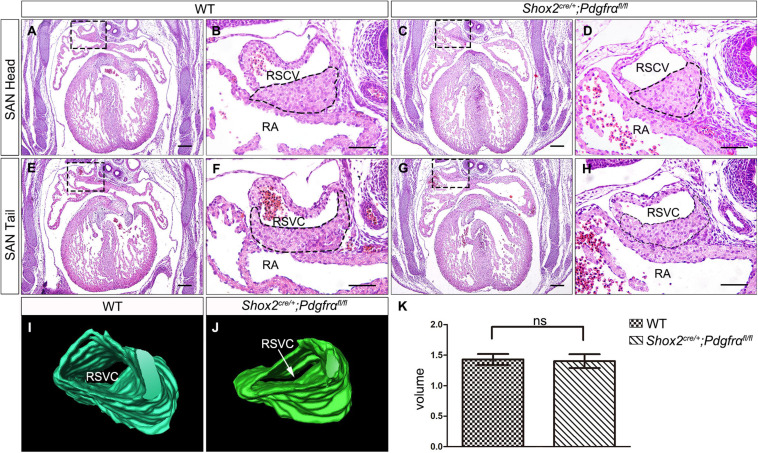
Formation of normal SAN in *Pdgfrα* conditional knockout mice. **(A–H)** H&E staining reveals the SAN with normal morphological characteristics in the E13.5 *Shox2-Cre;Pdgfrα^*flox/flox*^* developing heart as compared to the wild-type. **(I,J)** 3D reconstruction of the SAN from E13.5 wild-type and mutant heart. **(K)** Statistical analysis based on 3D reconstruction shows no significant difference in cardiac volume between *Pdgfrα* conditional knockout mice and controls. RA, right atrium; RSVC, right superior vena cava. Scale bar = 50 μm.

To further investigate the role of *Pdgfrα* during SAN development and avoid embryonic lethality, we next generated a conditional knockout mouse line *Shox2-Cre;Pdgfrα^*flox/flox*^*, which exhibit Cre activity in the sinus venosus as early as E9.5 (Sun et al., 2013). To validate the efficiency of Cre recombinase in inactivating floxed gene, we crossed *Shox2-Cre* mice to Cre reporter *Rosa26*^*mTmG*^ allele. Immunostaining for GFP and Pdgfrα showed that *Pdgfrα* was clearly present in the sinus venosus of E9.5 *Shox2^*Cre/*+^*;*Rosa26*^*mTmG*^ control embryos (Figures 2A,C) but absent from that of the mutant ones (Figures 2B,D), confirming a successful knockout of *Pdgfrα* in the sinus venosus of *Shox2-Cre*;*Pdgfrα^*flox/flox*^* mice at this early stage. But to our surprise, analysis of electrocardiogram (ECG) in adult *Shox2-Cre;Pdgfrα^*flox/flox*^* mice showed that the electrocardiograms are normal with the P waves corresponding to atrial depolarization clearly identical to their adult wild-type littermates and each P wave followed by a normal QRS complex corresponding to ventricular depolarization (Figures 2E,F; *N* = 6/each group). This normal electrocardiogram from *Shox2-Cre;Pdgfrα^*flox/flox*^* mice indicated that cardiac function, particularly the pacemaking function of the SAN, was normal in the *Pdgfrα* conditional knockout mice. Given the fact that a smaller SAN was acquired with 37% reduction in volume at E13.5 in *Pdgfrα* conventional knockout mice in the previous study (Bax et al., 2010), we further performed morphological analysis and 3D reconstruction of the SAN from E13.5 mutant embryos. As shown in Figure 3, we did not find any morphological malformation and volume difference in the mutants as compared to their littermate controls (Figure 3, *N* = 3/each group). Therefore, our results provided compelling evidence that *Pdgfrα* is dispensable for SAN morphogenesis and physiological function in mice when it is ablated with Shox2-Cre at E9.5.

### Unaltered Expression of *Nkx2.5* in the SAN of *Shox2-Cre*; *Pdgfrα^flox/flox^* Mice

**FIGURE 4:**
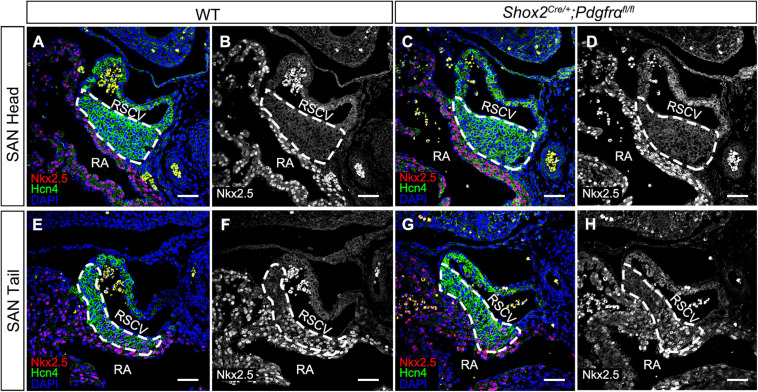
Unaltered *Nkx2.5* expression in the SAN of *Pdgfrα* conditional knockout mice. **(A–D)** Negative staining of Nkx2.5 in the SAN head, marked by Hcn4, of the E13.5 wild-type and mutant heart. **(E–H)** A similar expression pattern of Nkx2.5 is detected in the SAN junction, marked by Hcn4, of the E13.5 mutant heart as compared to the wild-type. RA, right atrium; RSVC, right superior vena cava. Scale bars = 50 μm.

**FIGURE 5:**
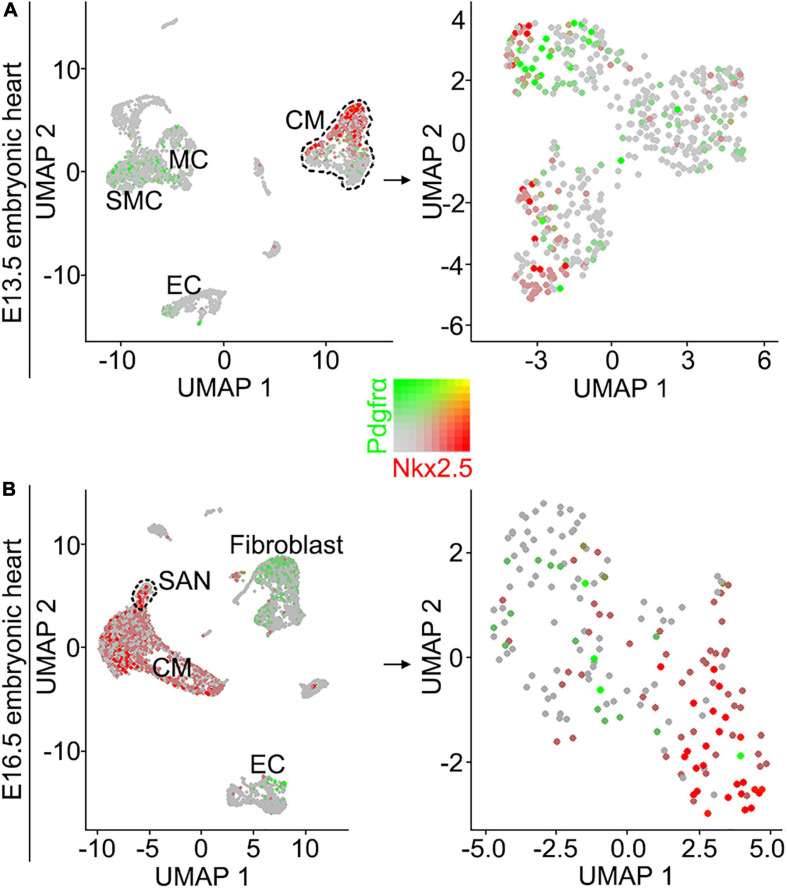
Analysis of scRNA-seq data reveals that *Pdgfrα* and *Nkx2.5* are not co-expressed in the same individual cells of the E13.5 and E16.5 SAN. **(A)** UMAP plots showing the distribution of *Pdgfrα* (green) and *Nkx2.5* (red) in the eight defined clusters (left) and three subset of the CM cluster (right) on the SAN and atrial cells from E13.5 *Shox2*^*cre/*+^*;R26R*^*mTmG*^ mice. **(B)** UMAP plots showing the distribution of *Pdgfrα* and *Nkx2.5* in the eight defined clusters (left) and the subset of SAN cluster (right) on the SAN and atrial cells from E16.5 WT mice. SMC, smooth muscle cells; MC, mesenchymal cells; CM, cardiomyocyte; EC, endothelial cells.

Although the SAN is a heterogenetic tissue with an Nkx2.5^–^ head and an Nkx2.5^+^ junction (Christoffels et al., 2006; Ye et al., 2015a), the fact that overdosed Nkx2.5 in the head led to severe hypoplasia of the SAN and that the mice with *Nkx2.5* deletion in SAN show normal SAN morphogenesis but SAN dysfunction indicates that Nkx2.5 activity is detrimental to formation of the SAN (Espinoza-Lewis et al., 2011; Li et al., 2019). Prior study showed the formation of a significant smaller SAN in conventional *Pdgfrα* knockout mice was accompanied with increased *Nkx2.5* expression (Bax et al., 2010), while our results showed formation of the SAN with normal morphology and physiological function in *Shox2-Cre;Pdgfrα^*flox/flox*^* mice (Figure 3), we reasoned that the expression of *Nkx2.5* would be unaltered in the SAN of the mutant mice. We, therefore, subsequently examined the expression of *Nkx2.5* in the SAN of *Shox2-Cre;Pdgfrα^*flox/flox*^* mice. Our immunostaining showed that, while there is no Nkx2.5 present in the SAN head marked by Hcn4 (Figures 4A–D), *Nkx2.5* is indeed expressed in the junction of the mutants in the pattern similar to that of the wild-type one (Figures 4E–H; Wiese et al., 2009; Liang et al., 2013), indicating unaltered expression of *Nkx2.5* in the SAN of *Shox2-Cre;Pdgfrα^*flox/flox*^* mice compared to the controls. Thus, our results demonstrated that the lack of *Pdgfrα* posed no evidence of ectopic or enhanced expression of *Nkx2.5* in both the head and junction of the SAN. In consistent with this observation, analysis of scRNA-seq data from the E13.5 SAN revealed that, of the eight visualized clusters (Li et al., 2019), *Pdgfrα* was present in smooth muscle cells (SMC), mesenchymal cells (MC), endothelial cells (EC) and cardiomyocytes (CM), but *Nkx2.5* was only restricted in CM and appeared not overlapped with *Pdgfrα* (Figure 5A-left). Further analysis of the cells in the CM cluster identified three clusters, in which the expression of *Pdgfrα* and *Nkx2.5* was indeed not co-expressed in the same individual cells (Figure 5A-right). This non-co-expression pattern of *Pdgfrα* and *Nkx2.5* in the same individual cells of the SAN was further confirmed with the analysis of scRNA-seq data from the E16.5 SAN (Figure 5B). Obviously, Pdgfrα, as a membrane-residing signaling molecule, would not exert its regulatory function on the cells where it is not expressed.

## Discussion

As a heterogeneous structure, the SAN is composed of a head and junction and several specialized cell types including myocardial pacemaker cells and a substantial number of non-pace maker cells. At E8.0-8.5, the dominant pacemaking activity originates from the left inflow tract region (Yi et al., 2012). By heart looping stage, pacemaking activity is localized to the right inflow tract (Dyer and Kirby, 2009; Csepe et al., 2015). These cells are eventually restricted to the dorsal wall of the right atrium and become a morphologically discernible SAN at E10.5, which further proliferates and matures at E13.5 (Virágh and Challice, 1982). Lineage studies indicate that the Nkx2.5^–^ head and Nkx2.5^+^ junction are derived from distinct progenitor population and represent separate regulatory domain expressing distinctive gene programs at E7.5 to E8.5 (Liang et al., 2013; Später et al., 2013). Formation of the SAN is tightly regulated by a complex network comprized of various signaling molecules, their receptors, and the intracellular signal transduction and transcription system (Wiese et al., 2009; Munshi, 2012). Reciprocal interactions among cardiac progenitor cells, mediated *via* secreted growth factor activated signaling including Pdgfrα signaling, are critical players in establishment of such a network (Bax et al., 2010).

The expression of *Pdgfrα* can be observed in the anterior lateral plate mesoderm as early as E7.5 and in caudal the second heart field (SHF) progenitors and dorsal mesocardium at E8.0 (Prall et al., 2007; Bloomekatz et al., 2017). Its expression then identifies cardiac progenitor cells in the posterior part of SHF at E8.5 and can be found at the venous pole in the mesocardium, the sinus venosus myocardium at E9.5 in mouse (Van Den Akker et al., 2005; Bax et al., 2010). At E10.5 when SAN is morphologically discernible, the developing SAN is also positive for the receptor (Van Den Akker et al., 2005). Our immunostaining reveals that *Pdgfrα* remains apparently expressed until E13.5 when the SAN is becoming mature. Conventional deficiency of Pdgfrα signaling by targeted disruption of this gene in mice led to atrial and sinus venosus myocardium hypoplasia, including formation of hypoplastic SAN with 37% reduced volume and increased myocardial expression of *Nkx2.5* (Bax et al., 2010). However, in this study, we found that a SAN was formed with normal morphology and ECG in *Shox2-Cre;Pdgfrα^*flox/flox*^* mice where *Pdgfrα* was specifically ablated using *Shox2-Cre* allele.

Several Cre lines have been designed previously for cardiac-specific excision of loxP-flanked target genes (Chien, 2001; Hoesl et al., 2008; Arnolds and Moskowitz, 2011; Sun et al., 2013). Among them, Hcn4-CreER^*T2*^ allele represents the first tool for deletion of floxed genes selectively in the cardiac conduction system since *Hcn4* is expressed specifically in both the sinoatrial and atrioventricular node (Hoesl et al., 2008). In comparison with *Hcn4*, *Shox2* expression is restricted to the inflow tract and sinus venosus in the posterior heart field that give rise to the SAN and the sinus valves. Expression of *Shox2* initiates in a restricted pattern at the junction formed by the common cardinal vein, the common atrial chamber and the sinus venosus at E8.5, restricted to the forming sinus horns region at the junction between the common cardinal vein and the common atrium (the sinus venosus) at E9.5, extends to the sinus valves originating from the SAN at E10.5, and becomes restricted to the entire SAN including the head and tail regions at E11.5 and later (Espinoza-Lewis et al., 2009; Ye et al., 2015b). Moreover, Sun et al. (2013) constructed the Shox2-Cre knock-in mice, and they found the Cre activity was first detected in the developing sinus venosus at E9.5 and subsequently at E10.5 and E11.5 in all tissues with the expression of *Shox2*, including the SAN, the proximal domain of the limb, the anterior region of the palatal shelves, the temporomandibular junction, as well as the dorsal root ganglia and brain. These results indicate that the *Shox2-Cre* allele is another suitable *Cre* line to manipulate gene function in SAN development and the ablation of *Pdgfrα* with Shox2-Cre will specifically inactivate the Pdgfrα signaling in the tissue that give rise to the SAN and the sinus valve at not earlier than E9.5 when *Shox2* is expressed. Whereas, ablation of *Pdgfrα* in conventional knockout mouse line will inactivate Pdgfrα signaling in all tissue including cardiac progenitor cells and their derived tissues as early as E7.5. Given the importance of the interaction among cardiac progenitor cells and the fact that the production of electrical impulse by early pacemaker cells as early as E8.0-8.5, we believe that there is a cell population designated for the fate of SAN and their developmental fate has been determined at E8.5 when Shox2 is expressed. Pdgfrα signaling is crucial for commitment of these SAN progenitor cells, and once the fate of these cells are committed, Pdgfrα signaling is no longer required.

In addition, conventional deficiency of *Pdgfrα* results in increased myocardial expression of *Nkx2.5*, leading to formation of hypoplastic SAN (Bax et al., 2010). Nkx2.5 is a cardiac differentiation marker, which is expressed in the SAN junction (Espinoza-Lewis et al., 2009; Ye et al., 2015b). Although mice lacking *Nkx2.5* in the SAN junction adopt normal morphology, the physiological function is impaired, indicating Nkx2.5 is dispensable for SAN morphogenesis but required for physiological function. Formation of hypoplastic SAN caused by overexpression of *Nkx2.5* in the heart and overdosed Nkx2.5 in the SAN head when Shox2 repressive function is deficient further supports the idea that overdosed Nkx2.5 is detrimental to SAN morphogenesis (Espinoza-Lewis et al., 2009, 2011; Liu et al., 2012). Our results showed that *Nkx2.5* expression in *Pdgfrα* conditional knock-out mice was unaltered and that *Pdgfrα* and *Nkx2.5* were not co-expressed in the same individual cells of the mature SAN, suggesting that Pdgfrα signaling exerts only its early function on repression of *Nkx2.5* during development of posterior heart field derived cardiac structures (Bax et al., 2010).

## Data Availability Statement

The datasets presented in this study can be found in online repositories. The names of the repository/repositories and accession number(s) can be found in the article/supplementary material.

## Ethics Statement

The animal study was reviewed and approved by The Animal Ethical and Welfare Committee of Fujian Normal University.

## Author Contributions

XuH designed the study and administrated the whole study. XZ and FW conducted experiments and collected data and drafted the manuscript. HL provided the E13.5 SAN scRNA-seq raw data. XiH analyzed the scRNA-seq data. ZG involved in methodology and statistical analysis. YZ and XH performed manuscript reviewing and polishing. All authors gave final approval of the version to be published, and agreed to be accountable for all aspects of this work.

## Conflict of Interest

The authors declare that the research was conducted in the absence of any commercial or financial relationships that could be construed as a potential conflict of interest.
